# Thomas Dover: Physician and Merchant Adventurer

**Published:** 1909-03

**Authors:** J. A. Nixon

**Affiliations:** Physician to the Bristol Royal Infirmary


### THOMAS DOVER: PHYSICIAN AND MERCHANT ADVENTURER.

BY

J. A. Nixon, M.B. Cantab., M.R.C.P.,
*Physician to the Bristol Royal Infirmary.*

In a recent review of a volume of essays by Professor Osler[1] Thomas Dover was referred to as "Dover the Buccaneer, who for four years harassed the coast of South America; . . . thoroughness pervaded both his medicine and his piracy." Thus do our simplest actions suffer misconstruction at the hands of posterity. Here is a conception of Dover sailing the Spanish Main steeped in the blood of hecatombs of innocent mariners, genially encouraging the timorous hearts to step out boldly along the plank to sup with Davy Jones, and paying his morning and evening devotions to the "Jolly Roger" at his masthead. The truth, however, reveals a much more prosaic personality. The expedition on which Thomas Dover sailed was in no sense of a piratical nature ; it was one of those adventurous trading cruises of which Hakluyt in his *Principal Voyages of the English Nation* gives many instances. It was an accident of the times that few of these trading expeditions ran to their finish without the spilling of blood, for in 1708 the power of Spain was not yet broken in America and the Indies, while France, emboldened by the decline of the Dutch navy, was making free use of the high seas. Small wonder if an English merchant vessel set out ready to fight if need be, and show by her sharp teeth that she, too, claimed the right to pass on the seas upon her lawful occasions. Commerce did not follow the flag in those days, but bore it close company in its journeyings over distant lands and oceans.

The voyage round the world of the two privateers, the *Duke* and *Duchess*, under command of Captain Woodes-Rogers,[2] was the properly-accredited enterprise of the Merchant Adventurers of Bristol. The owners included three merchants who sooner or later were Mayors of Bristol, Sir John Hawkins (1701), James Hollidge (1709), and Christopher Shuter (1711), together with Captain Philip Freake and Thomas Clements, who were sheriffs, and John Romsey, town clerk.[3] These and several others contributed the necessary expenses of the expedition, and it was in virtue of his own share that Dr. Dover was selected to represent the owners' interests, and sailed on the *Duke* as second captain under Woodes-Rogers, and captain of the marines. The navigation of the sister ships was entrusted to Woodes-Rogers : but Dover was appointed to preside over all the councils, with the privilege of exercising two votes ; and his share of all plunder was calculated in thousands as against the hundreds of the other senior officers, indicating that he was interested financially in a higher ratio than any others on board. The council for directing the affairs of the ships *Duke* and *Duchess* (*sic*) in their voyage to America was composed of Captain Woodes-Rogers, commander, Captain

Thomas Dover, and Captain Dampier, pilot, with other officers. "In case of equality Captain Dover is to have double voice as President of the Council."

The medical welfare of the ships should have been amply provided for. In addition to Dover, there sailed "John Ballet, rated third mate, but designed surgeon if occasion (he had been Captain Dampier's doctor in his last unfortunate voyage round the world); Samuel Hopkins, being Dr. Dover's kinsman* and apothecary, was both assistant to him and to act as his lieutenant if we landed a party anywhere under his command during the voyage;" John Vigor, a reformado, to act as Captain Dover's ensign when ashore; James Wasse, surgeon, Charles May, his mate, and John Lancy, assistant. Another Hopkins (William) was designated ship's corporal, Captain Dover's serjeant, and cook to the officers. Yet in spite of this array of surgeons and assistants, Woodes-Rogers has reason to record: "Our surgeons make heavy complaints for want of sufficient medicines with which till now I thought we abounded, having a regular physician, an apothecary and surgeons enough, with all sorts of medicines, on board . . . . but now we found it otherwise."

It appears that Dr. Dover's interest in the undertaking was commercial rather than medical. On another occasion a doubt arises as to the technical skill of the surgeons, for on October 30th, 1709, it is recorded: "One of our negro women cryed out, and was delivered of a girl of tawny colour. Mr. Wasse, our chief surgeon, was forced to discharge the office of midwife," in which duties, it seems, he was somewhat unpractised.

Dover made himself, as was his wont, an extremely cantankerous travelling companion, and eventually, on October 11th, 1709, was persuaded to betake himself to the other ship, the *Duchess*.

In the January following he asserted his seniority in a fashion little to the liking of the ships' officers. A prize had been taken, and Dover claimed as second captain to be appointed to command her, but the navigating officers protested: "We therefore (being inclined to peace and quietness aboard, and not to use any violence

* He had married Dover's sister.

to remove the said Captain Dover out of the aforesaid forc'd command, although he is utterly incapable of the office) do hereby publickly protest against the aforesaid commander." Even as a buccaneer he seems to have had his limitations. Woodes-Rogers, in answer to this protest, writes: "I being very weak and in much pain [he had been shot through the cheek] was not able to stir, therefore sent my opinion in writing as follows, 'My opinion is, that 'tis not for the safety of the rich Spanish prize that Captain Dover command her, because his temper is so violent, that capable men cannot well act under him, and himself is uncapable!'" Ultimately he sailed as commander of the prize, but the honour was purely titular, for he was forbidden to interfere in any way with the navigation of the ship, a competent captain being appointed who was to take no orders from him. Presumably, he accepted the position with such grace as he could exhibit, for the expedition came safe to port.

That his share of the booty was substantial may be judged from the fact that in 1712 John Romsey presented to the Bristol Cathedral a pair of large silver candlesticks of the plunder in this expedition which cost him £114.[4] These candlesticks,* by the way, were afterwards removed, but were restored in 1891, soon after the death of Dean Elliott. Romsey was town clerk in 1685, at the assize when Judge Jeffreys made his well-known comments on the kidnapping propensities of the civic magnates of Bristol.

It was, in all likelihood, Dover's violent temper that earned him the cordial dislike of the medical profession of his day, quite as much as his advocacy of quicksilver.

As a physician he had no little experience, and was well-qualified to practise medicine. Like his master, Sydenham, he migrated from Oxford to Cambridge in order, doubtless, that he might sit at the feet of Dr. Brady, then Master of Gonville and Caius College. The register of admissions shows that at Michaelmas, 1686, the first to enter at that college was "Thomas Dover, from St. Mary Hall, Oxford, where he resided six years, admitted pensioner to the scholars' table. Surety, Mr. Lightwine.

* They are of English manufacture, and bear the hall-mark of Queen Anne's reign.

Matriculated at Magdalen Hall, Dec. 1, 1680. Age 16. Son of John Dovẽr, of Barton-on-the-Heath, Warwicks, gent. B.A. Oxford, 1684. M.B. (Cambridge), 1687."[5]

At a later date he settled to practise in Bristol. "The first medical man who gratuitously offered his services on behalf of the poor under the care of the guardians of this city was Dr. Thomas Dover in 1696. This gentleman, in 1708, became part owner and second captain of the *Duke*, privateer, under the command of Captain Woodes-Rogers, and went with him upon a cruise into the South Seas. The Doctor, now Captain Dover, went ashore on the Island of Juan Fernandez, where he met with Alexander Selkirk, a Scotchman, who had lived there four years and four months, having been left on that uninhabited island by Captain Stradling of the *Cinque Ports*. Selkirk, upon his return to England, gave his papers to Daniel Defoe, who manufactured therefrom the interesting and well-known history of *Robinson Crusoe*. Dr. Dover, towards the decline of his life, resumed his profession, and made much noise in the medical world by recommending the use of crude mercury," but says Johnson,[6] writing in 1828, "I am not informed if Bristol then became his residence." Dover himself describes his work on behalf of the poor under the guardians: "About fifty years since, this Fever raged much in Bristol [*i.e.* spotted fever], so that I visited from twenty to thirty patients a day for a considerable time, besides their poor children taken into their workhouse, where I engaged myself, for the encouragement of so good and charitable an undertaking, to find them Physick and give them advice at my own expence and trouble, for the two first years."[7]

Evidently Dover was a wealthy man who practised medicine because he liked it, and without much thought of profit. His buccaneering amounts to accompanying in person one of the numerous trading expeditions which set out from Bristol and other ports to obtain by purchase or persuasion the produce of the New World, and, if Fortune smiled, the rich cargoes of sundry foreign galleons.

The entry in the Caius College register of admissions has thrown considerable light on the hitherto unknown ancestry of

Thomas Dover. The present Rector of Barton-on-the-Heath, Rev. A. Nettleship, has very kindly searched the baptismal registers of his parish, and has found the following entry in the year 1662:—

"Thoma Douer filius Johañis Douer gen./et Elizabethæ uxoris ejus baptizatus sexto/die Maij An pdict."

Vyvyan,[8] in the introduction to his reprint of Captain Robert Dover's *Annalia Dubrensia*, gives a genealogical table of this branch of the Dover family, which he states is in the handwriting of the late Sir Thomas Phillipps, Bart., in the copy of the *Annalia* belonging to that well-known collector's library.

From this it would appear that Thomas was the grandson of Robert Dover, who founded the famous Cotswold Games, as related by Rudder[9] in his *History of Gloucestershire*:—

"Mr. Robert Dover, who lived in the reign of King James the First, became a very popular man in this country by his hospitality and generosity. He instituted an annual meeting for the practice of all sorts of manly exercises, and distributed prizes to such as excelled in them. These exercises and their patron are the subject of a small collection of verses intituled *Annalia Dubrensia*, written by the best poets of that age. And there is still (1779) a meeting of young people upon Dover's Hill, about a mile from Campden, every Thursday in Whitsun Week."

Robert Dover (1575–1641), an attorney "who never try'd but two causes, always made up the difference," was the son of John Dover, of Norfolk. He married Sibilla, daughter of Dr. Cole, Dean of Lincoln, and had two daughters and one son, Captain John Dover (b. 1614, æt. 68 in 1682), who fought under Prince Rupert. According to the Phillipps' genealogy, this John Dover married Elizabeth, daughter of Thomas Vade, of Barton-on-the-Heath, and had issue four daughters and three sons: Robert, who died in infancy; John, of Gray's Inn, æt. 38 in 1682, cœlebs; and Thomas, æt. 19 in 1682.

John, of Gray's Inn, figures in the Barton Registers in the year 1644: "John the sonne of John Dover gent/baptized the 28th of October." In *Dict. Nat. Biog.* he is described as a dramatist, the son and heir of John Dover, of Barton-on-the-Heath, and

John Dover, of Norfolk.

William Cole, Dean of Lincoln.

Robert Dover, of Barton-on-the-Heath = Sibilla, d. of — Cole,
Founder of the Cotswold Games, Dean of Lincoln, d. 1597.
ob. 1652, living 1622.

W[illegible]m =
Thomas.

Robert, ob. infant.

John, of Barton-on-the-Heath = Elizabeth, d. of Thomas Vade, co. Warwick, æt. 68, 1682, of Barton-on-the-Heath, born 1614. Baptised at by Joan, d. and heiress of -- Keck.

Seynbury, co. Gloucester, 1614.

Abigail = Anthony Stratford,
of co. Gloucester.

Sibilla = George Stratford.

Robert. ob. infant.

Mary=Samuel Hopkins.

Magdalen=Thomas Titford.

Sarah=Charles Stokes, of Banbury.

*(Issue omitted here.)*

Ann=John Leeson, of London.

**Thomas, æt. 19, 1682.**

Sibilla=(1) John Norden.

(2) — Cakebread.

John, of Gray's Inn, æt. 38, 1682, cœlebs.

(*From* Vyvyan.)

grandson of Robert Dover. In 1661 he was admitted demy at Magdalen College, Oxford, but left in 1665 without taking a degree. He intended to follow the law, and entered at Gray's Inn May 19th, 1664. Ultimately he took orders in the church, and became in 1688 Rector of Drayton, where he died in 1725 in his eighty-second year.

The evidence here agrees with the supposition that the Thomas Dover who matriculated at Magdalen Hall, Oxford, in 1680, at the age of sixteen, and entered Caius College in 1686, is the person referred to in the baptismal register of Barton, and in the Phillipps' genealogical table. A grave difficulty, however, arises out of a statement in the *Dict. Nat. Biog.* that the Rev. John Dover was born after his mother had passed her sixty-first year, so that she must have been seventy-eight when Thomas was born. This statement is given on the authority of Mrs. Cordwell, the daughter of the Rev. John Dover, and is on the face of it unlikely, since Captain John Dover was born in 1614, and was therefore only thirty when his son John was born in 1644. It is more reasonable to accept Sir Thomas Phillipps' account, for this is supported in other directions.

Thomas Dover clearly belonged to this family, and passed no small part of his life in the fair Cotswold country. He dedicated the *Physician's Legacy* to Robert Tracy, Esq., of Stanway, in Gloucestershire (where Robert Dover had built a house in which he died, and was buried in the parish church June 6th, 1641). In 1700 he issued a reprint of *Annalia Dubrensia*, in which, after the letter from Walbancke to Robert Dover, the following words occur: "Dr. Dover thought it his Duty to perpetuate the Memory of that Good Man his Grandfather." *

Vyvyan confuses this Dr. Dover with John Dover, the Rector of Drayton; but the latter never proceeded to a doctor's degree.

* I give this on the authority of Vyvyan and *Dict. Nat. Biog.* The British Museum has two editions of the *Annalia Dubrensia* printed in 1636 (Robert Dover and Walbancke), one later edition (without title-page, &c.) undated, and reprints in 1877 and 1878. Watt, Birt and Loundes in their respective books know of nothing republished in 1700.

Hyett, *Trans. B. and G. Arch. Soc.*, xiii, 179, deals fully but inconclusively with the authorship of this edition.

On the other hand, Rudder gives to Thomas Dover the credit of being the founder of the Cotswold Games when he describes Stanway Hall: "The present manor-house is large and handsome, situated on the slope of the hill, with good gardens, and a cascade of water, seen from the vale below at the distance of several miles. It was built by Sir Paul Tracy in the reign of King James the First, and was the seat and residence of Robert Tracy, Esq., till the time of his death in the year 1767. The famous Doctor Dover who instituted the Cotswold Games died at this house in the year 1742, and was buried at his own request in the vault belonging to the Tracy family. No gentleman now (1779) resides at Stanway House."

The same account occurs in Rudge's[1] *History of the County of Gloucester*, and is probably a quotation from Rudder: "Doctor Dover, well known for the institution of the Cotswold Games, died here (*i.e.* Stanway) in 1742, and at his own request was buried in the family vault of the Tracys." Both these historians depended very largely for their information on Sir Robert Atkyn's *Ancient and Present State of Gloucestershire* (1712); but as no statement akin to this occurs, it is not a misquotation from Atkyns. Rudder is responsible for originating the story, and it should be noted that he gives the date of Thomas Dover's death as 1742, thus agreeing with other authorities. The statement in the *Dict. Nat. Biog.* and elsewhere, that Dr. Dover died in London at his house in Arundel Street, is based upon a conjecture of Munk's.[11] The accuracy of the Gloucestershire historians cannot well be impugned on the strength of such a conjecture, and there can be little doubt that in his own Cotswold home Thomas Dover, M.B., was better known and better loved than in London, where, as "the quicksilver doctor," the hot-tempered circumnavigator met with scant courtesy from "the gentlemen of the Faculty who," he says, "like moles, work underground, lest their practices should be discovered to the Populace."

### REFERENCES.

1 **Osler,** "An Alabama Student, and other Biographical Essays," *St. Barth. Hosp. J.*, 1908, xvi, 29 (review).

2 **Woodes-Rogers,** *A Voyage round the World* (commonly known as the Bristol Privateer).

[3] **Latimer,** *Eighteenth-Century Bristol,* 1893, p 74.
[4] **Evans,** *Chronological History of Bristol.*
[5] **Venn,** *Biographical History of Gonville and Caius College.*
[6] **Johnson,** *Bristol Corporation of the Poor.*
[7] **Thomas Dover, M.B.,** *The Ancient Physician's Legacy to his Country* (seventh edition, p. 107).
[8] *Annalia Dubrensia* (Vyvyan, 1878).
[9] **Rudder,** *New History of Gloucestershire* (1779).
[10] **Rudge,** *History of the County of Gloucester* (1803).
[11] **Munk,** *Roll of the College of Physicians.*

